# Anthranilate Reduces *Pseudomonas aeruginosa* Infection Severity Through an Antibiotic-Independent Anti-Virulence Mechanism

**DOI:** 10.3390/antibiotics15090898

**Published:** 2026-09-11

**Authors:** Huiyan Li, Min-Hee Kang, Wen-Xin Niu, Eunsoo Kim, Joon-Hee Lee

**Affiliations:** 1 College of Pharmacy, Pusan National University, Busan 46241, Republic of Korea; lihy0522@naver.com (H.L.); byj04046@naver.com (M.-H.K.); niuwenxin@naver.com (W.-X.N.); eunsoo_kim@pusan.ac.kr (E.K.); 2 Research Institute for Drug Development, Pusan National University, Busan 46241, Republic of Korea

**Keywords:** *Pseudomonas aeruginosa*, anthranilate, anti-virulence agent, quorum sensing, mouse skin infection, mouse pulmonary infection

## Abstract

Background/Objectives: Antibiotic resistance is a growing global health challenge, especially with tricky bacteria like *Pseudomonas aeruginosa*. Anthranilate, previously identified as a signaling molecule in *P. aeruginosa*, modulates various pathogenicity-related phenotypes by reducing virulence factor production, inhibiting biofilm formation, and increasing antibiotic susceptibility. Based on these good properties as an anti-virulence agent, this study evaluated the actual therapeutic potential of anthranilate using mouse skin infection and lung infection models. Specifically, the efficacy of anthranilate in a standalone treatment was investigated without reliance on conventional antibiotics. Methods: The therapeutic efficacy of anthranilate was evaluated in murine skin and lung infection models of *P. aeruginosa*. Anthranilate was administered as a standalone treatment, and its effects on bacterial burden, inflammatory responses, and tissue lesion progression were assessed and compared with those of gentamicin treatment. Results: Anthranilate effectively reduced bacterial suppuration, mitigated inflammatory responses, and suppressed lesion progression in infected skin and lung tissues. Notably, anthranilate alone produced therapeutic effects comparable to those of gentamicin. Conclusions: These findings highlight its potential of standalone treatment as an alternative to traditional antibiotics and offer a novel anti-virulence strategy that minimizes selective pressure for resistance development.

## 1. Introduction

*Pseudomonas aeruginosa* is one of the most formidable opportunistic pathogens that can cause a wide range of infections, including severe conditions such as bacteremia, sepsis, and ventilator-associated pneumonia, as well as clinically important skin and soft tissue infections. *P. aeruginosa* is known for its remarkable antibiotic resistance and robust biofilm-forming ability [1]. The increasing prevalence of multidrug-resistant (MDR) *P. aeruginosa* strains poses a severe threat to public health [2]. These MDR strains can resist multiple classes of antibiotics through diverse mechanisms such as efflux pumps, β-lactamases, and altered membrane permeability [3]. Moreover, biofilm formation provides an additional protective layer against both antibiotics and host immune responses, contributing to chronic infections and treatment failures [4]. *P. aeruginosa* also employs sophisticated quorum sensing (QS) systems that regulate various virulence factors including pyocyanin production, elastase secretion, and biofilm development [5]. The QS network in *P. aeruginosa* coordinates collective bacterial behaviors through multiple interconnected signaling pathways, making infections particularly difficult to control [6,7]. This complex interplay between QS, biofilm formation, and antibiotic resistance underscores the urgent need for alternative therapeutic strategies targeting these interconnected pathogenic mechanisms [8].

Efforts to develop new antibiotics have invariably encountered the persistent challenge of antibiotic resistance. In order to meet this problem, recent studies have highlighted the potential of “anti-virulence” compounds as promising alternatives or adjuvants to conventional antibiotics [9,10,11]. Anti-virulence drugs specifically suppress pathogenicity without impairing bacterial viability or imposing selective pressure, which means that they do not trigger resistance mechanisms. Instead, they change pathogens to a state more similar to non-pathogenic bacteria, preventing the progression of infectious diseases [12,13]. This novel therapeutic strategy holds promise for significantly reducing the emergence of resistance [14]. The concept of anti-virulence has been considered attractive and clever, and while several compounds that fit this concept have been reported, there are still relatively few cases where the efficacy of such anti-virulence agents has been validated in clinical settings or animal infection models.

Anthranilate has recently been suggested as a particularly intriguing anti-virulence candidate [15]. Anthranilate is a substance naturally produced by *P. aeruginosa* that has garnered significant attention due to its dual important roles. It serves as a precursor for PQS (*Pseudomonas* quinolone signal), a representative QS signaling molecule, and is also a precursor in the biosynthesis of the essential amino acid tryptophan [16]. On the other hand, anthranilate is also a breakdown product of tryptophan and can be metabolized via the TCA cycle, functioning as a nutrient source that directly supports *P. aeruginosa* growth [16,17]. During *P. aeruginosa* growth, anthranilate forms a characteristic concentration peak—the “anthranilate peak”—that functions as a signaling molecule influencing various physiological characteristics, particularly virulence-related phenotypes such as virulence factor production, biofilm formation, and antibiotic susceptibility [18]. Previous studies have demonstrated that elevated anthranilate concentrations can suppress biofilm formation and virulence factor expression in *P. aeruginosa* while simultaneously enhancing antibiotic susceptibility [18,19,20,21]. Notably, anthranilate achieves this without affecting the minimum inhibitory concentration (MIC) of antibiotics, suggesting that it suppresses antibiotic tolerance rather than antibiotic resistance [18]. In addition, anthranilate did not inhibit the growth of *P. aeruginosa* at all and did not show toxicity to animal cells even at a high concentration of 10 mM [20]. These results suggest that anthranilate can act as a good anti-virulence agent.

These anti-virulence characteristics of anthranilate hold significant clinical implications. By attenuating bacterial virulence and pathogenicity, anthranilate could potentially reduce infection severity as a standalone therapeutic agent [22]. Alternatively, it could increase susceptibility to antibiotics, thereby enhancing the efficacy of the antibiotics when used in combination. In addition, by targeting virulence rather than bacterial growth, it could minimize the selective pressure driving resistance development [23]. Therefore, this study aims to evaluate anthranilate’s potential as an antibiotic alternative in *P. aeruginosa* infection models through mouse experiments.

## 2. Materials and Methods

Bacterial strains and culture conditions. PAO1, a *P. aeruginosa* wild type strain, was employed in this study. PAO1 cells were cultured in Luria-Bertani [24] medium (0.5% yeast extract, 1% bacto-tryptone, and 0.5% NaCl) with vigorous shaking at 37 °C. For solid media, agar was supplemented at 1.5% (*w*/*v*). Bacterial growth was monitored by measuring optical density at 600 nm (OD_600_). For mouse infection studies, PAO1 cells were cultivated with vigorous agitation at 37 °C until reaching an OD_600_ of 3.0, subsequently harvested by centrifugation, and resuspended in sterilized phosphate-buffered saline (PBS). The two primary therapeutic agents used in this study, anthranilic acid (AA) and gentamicin [25], were purchased from Sigma-Aldrich (St. Louis, MO, USA) and Duchefa Biochemie (Haarlem, The Netherlands), respectively. All chemicals were used as received from the commercial suppliers without any further purification.

Animal experiment. To minimize order effects, the sequence of treatments was randomized for each subject. Additionally, we used a crossover design with sufficient washout periods between treatments to prevent carry-over effects. No specific inclusion or exclusion criteria were established a priori, as all subjects and data points were included in the final analysis. No animals were removed, and no outliers were excluded during the study. The experimental unit was defined as the individual animal. Due to the nature of the experimental setup and limited personnel, the researchers were aware of the group allocation at all stages including the allocation, the conduct of the experiment, outcome assessment, and data analysis.

**Figure 1:**
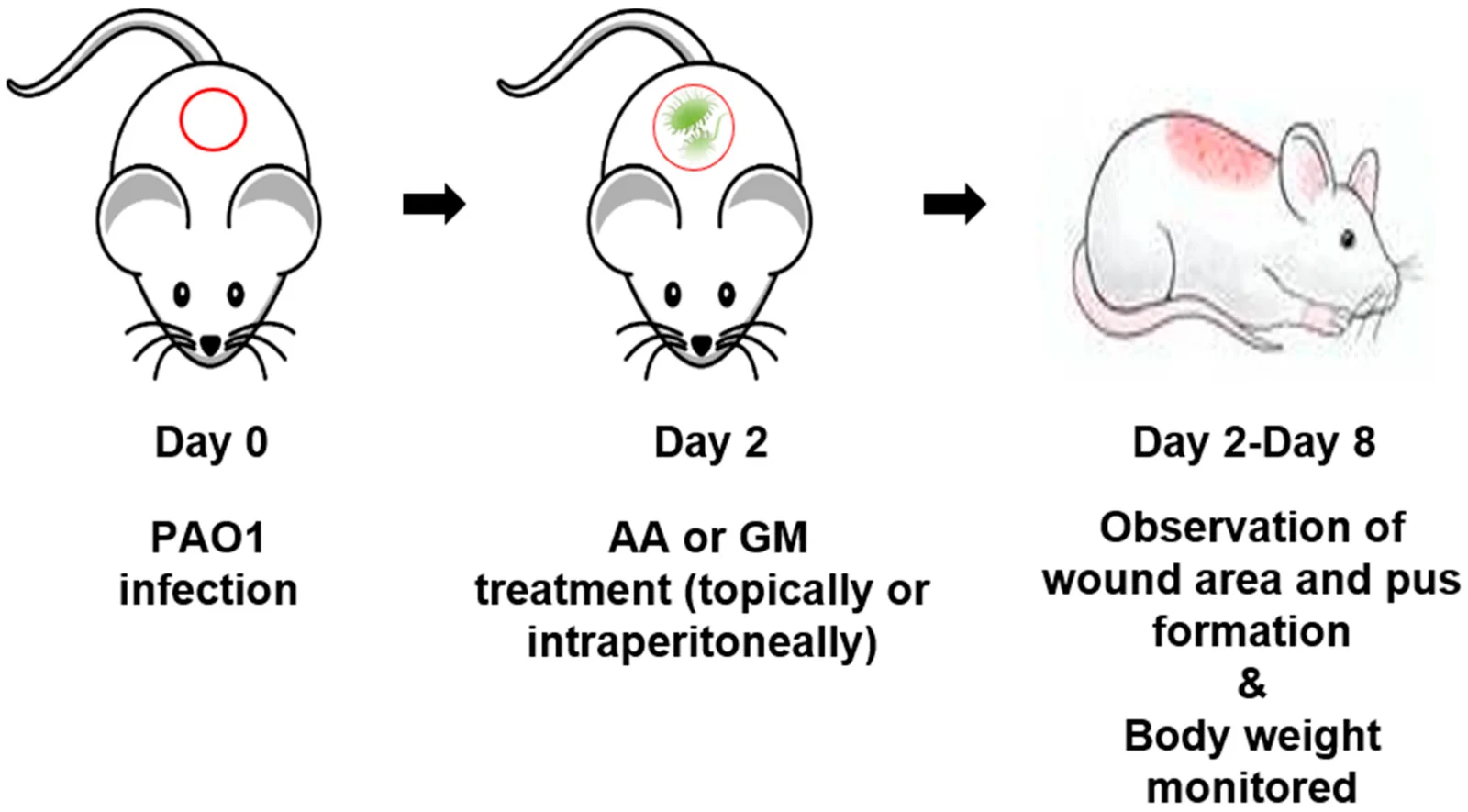
Experimental procedure for mouse skin infections with *P. aeruginosa*. Male Jcl:ICR mice were anesthetized for 20 min with avertin and the dorsal skin was shaved. Circular wounds (6 mm diameter) were created using a biopsy punch and 5 × 10^6^ CFU of *P. aeruginosa* were dropped onto the wound sites. After 2 days, when pus began to form at the site of infection, 2.74 mg/kg of anthranilic acid (AA) and/or 7.5 mg/kg of gentamicin [25] were dropped on the wound site or injected into the abdominal cavity by syringe. Wound areas were monitored and photographed every 2 day for 8 days.

**Figure 2:**
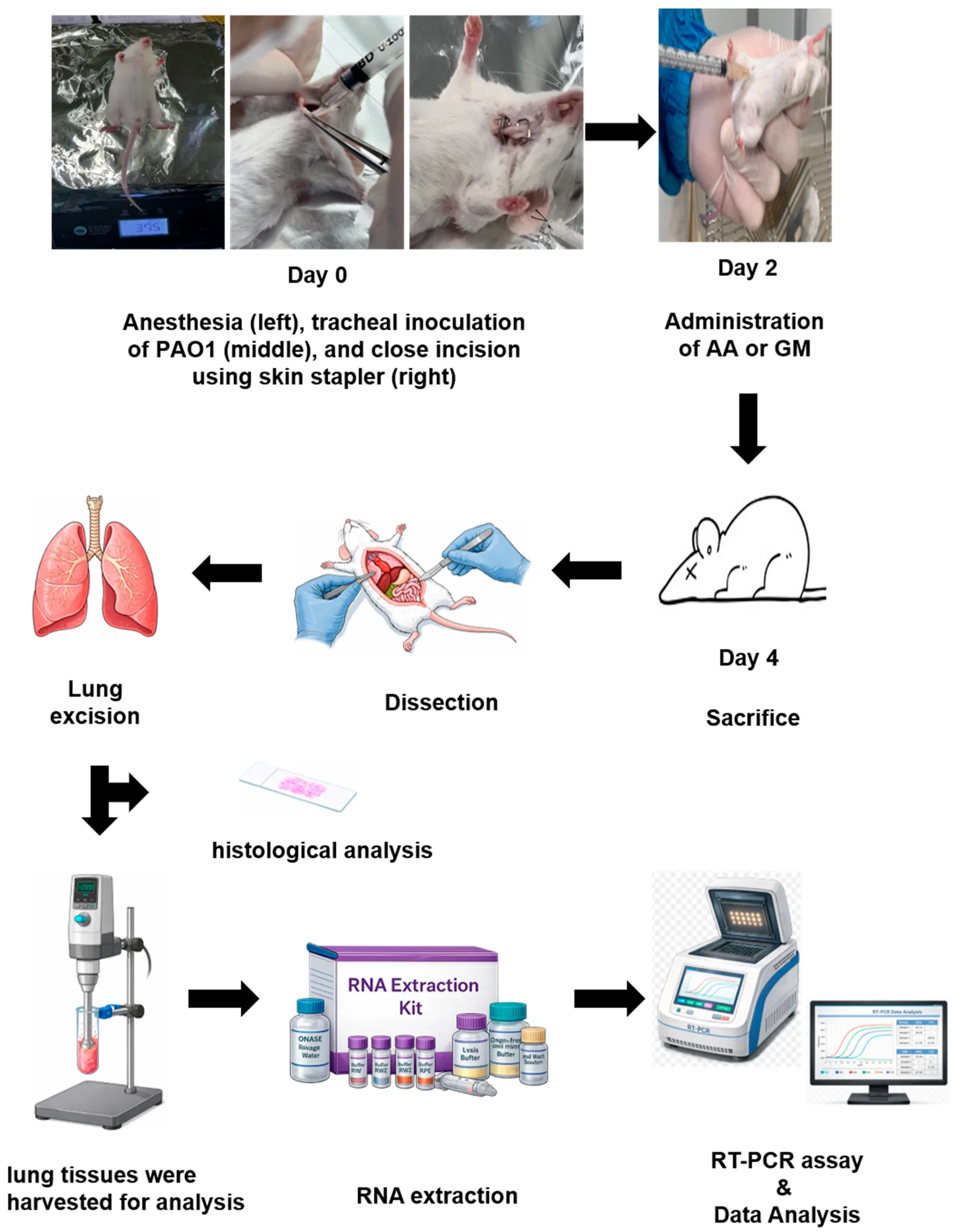
Experimental procedure for mouse lung infections with *P. aeruginosa*. Male Jcl:ICR mice were anesthetized and 2 × 10^6^ CFU bacterial cells were directly injected into the trachea through a neck incision, which was subsequently closed with a skin stapler. After 2 days, 7.5 mg/kg GM and 2.74 mg/kg AA were administered intraperitoneally. Mice were euthanized 48 h after treatment, and lung tissues were harvested for analysis.

**Figure 3:**
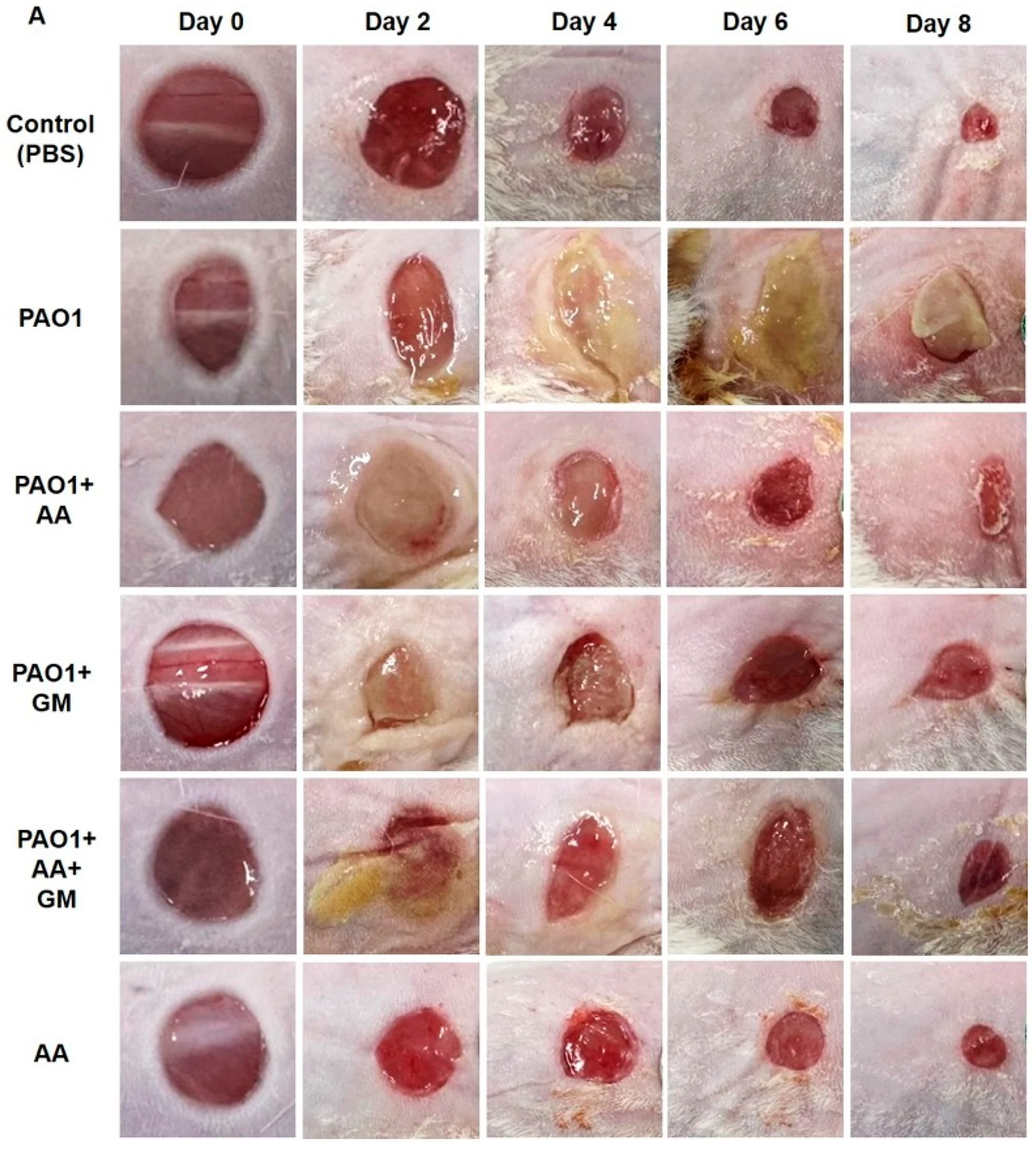

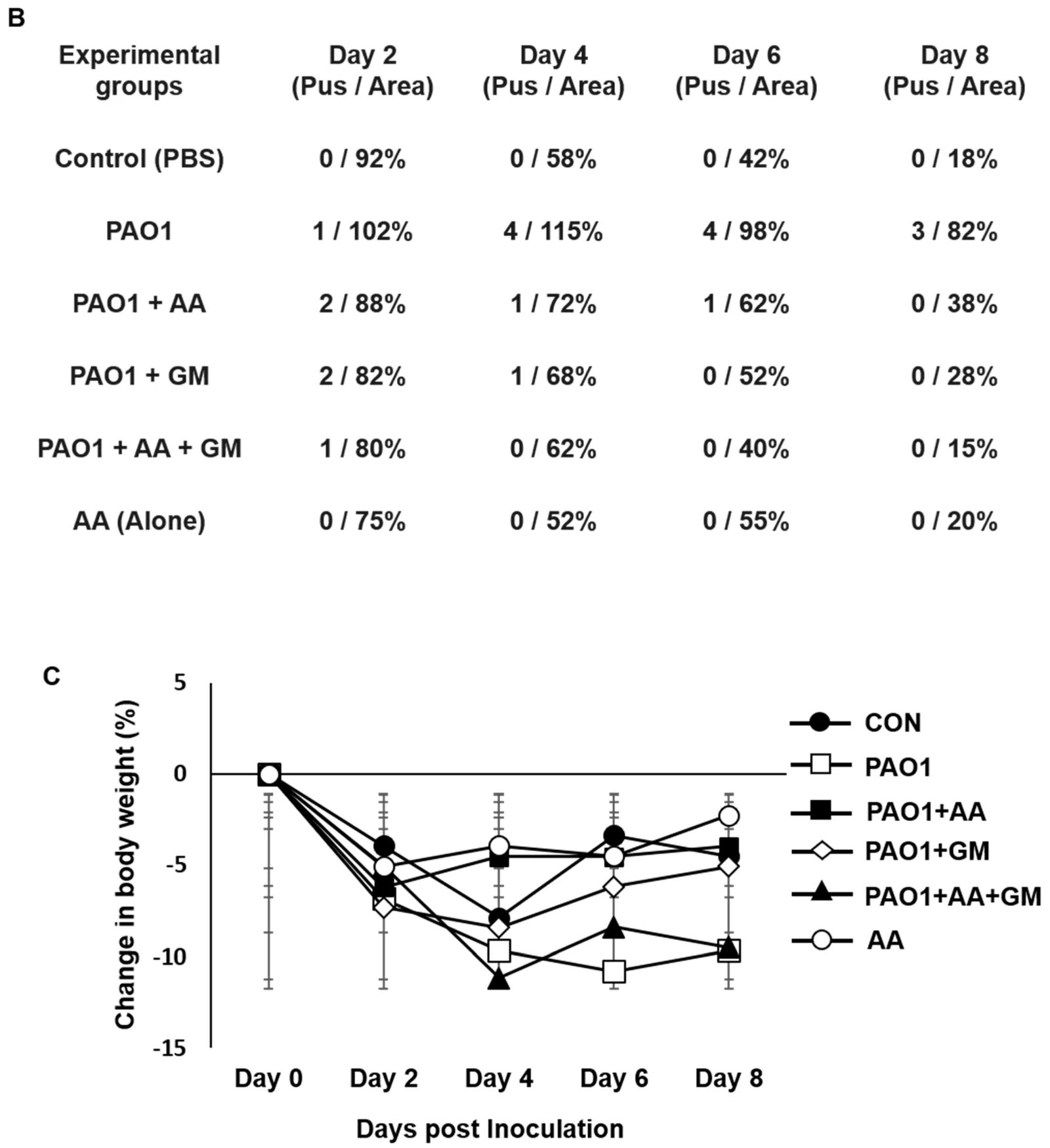
The effects of anthranilate on mouse skin infections—topical application. (**A**) The mouse skin infection experiments were conducted as described in Figure 1 in six groups as follows: Bacteria-free control and no treatment (PBS), *P. aeruginosa* infection but no treatment (PAO1), *P. aeruginosa* infection and anthranilic acid (AA) treatment (PAO1 + AA), *P. aeruginosa* infection and gentamicin [25] treatment (PAO1 + GM), *P. aeruginosa* infection and AA + GM treatment (PAO1 + AA + GM), and AA treatment without infection (AA). AA (2.74 mg/kg) and/or GM (7.5 mg/kg) were dropped on the wound site in 25 µL at 2 days after infection. *n* = 4–5 per each experimental group. (**B**) For the quantitative assessment of wound healing and infection severity in (**A**), wound progression was quantified using two distinct metrics: semi-quantitative pus scoring (Pus) and relative wound area (Area). The pus score was determined based on a standardized 5-point rubric (0–4). The relative wound area (%) represents the wound size at each time point (Day 2 to Day 8) normalized to the initial wound area on Day 0 (100%). (**C**) During the skin infection experiment, body weights were measured for 8 days following the infection and expressed as a percentage change from the initial weight. Data are presented as mean ± SD. *n* = 4–5 per each experimental group.

Mouse skin infection. The mouse skin infection experiment was performed as previously described [26], and the detailed experimental procedure is illustrated in Figure 1. Male Jcl:ICR mice (7 weeks old, Samtako Bio, Osan, Republic of Korea) were housed in polycarbonate cages (4–5 mice per cage) under controlled conditions (24 °C, 55% humidity, 12-h light/dark cycle). Mice were randomly assigned to six experimental groups—PBS, PAO1, PAO1 + AA, PAO1 + gentamicin, PAO1 + AA + gentamicin, and AA (*n* = 4–5 per group). After 20 min of anesthesia with 1.2% avertin (2,2,2-tribromoethanol; 20 mL/kg, intraperitoneal administration), the dorsal skin was shaved and disinfected with 70% ethanol. A circular full-thickness wound (6 mm in diameter) was created using a biopsy punch (Kai Medical, Seki-shi, Japan). Subsequently, 5 × 10^6^ CFU of *P. aeruginosa* PAO1 in 25 µL PBS was applied to the wound site. Control mice received bacteria-free PBS or anthranilic acid (AA) alone. Two days post-infection, when visible signs of pus formation were observed, mice received treatment according to group allocation. AA (2.74 mg/kg) and/or gentamicin (GM, 7.5 mg/kg) were administered either topically to the wound site in 25 µL (Figure 2) or intraperitoneally in 600 µL (Figure 3), depending on the experimental design. Wound areas were monitored and photographed every 2 days for 8 days (see Figure 1 for the entire procedure). The wound area was quantified using digital planimetry and expressed as a relative percentage of the initial wound area on Day 0 (100%). In addition, a semi-quantitative pus score was assigned based on a standardized 5-point rubric to assess the severity of suppuration and necrosis: Score 0 (clean wound, no discharge), Score 1 (mild serous exudate), Score 2 (moderate pus covering < 50% of the area), Score 3 (extensive suppuration > 50% with visible necrosis), and Score 4 (severe necrosis with thick, malodorous discharge). To minimize subjective bias, all scoring and area measurements were performed through the consensus of two independent researchers blinded to the experimental groups and further validated by two separate AI-based image analysis tools.

Mouse lung infection. The mouse lung infection experiment was performed as previously described [26], and the experimental procedure is illustrated in Figure 2. Briefly, 7-week-old male Jcl:ICR mice were randomly assigned to 6 experimental groups (*n* = 4–5 per group) like in the skin infection experiment above and anesthetized as described above. Mice were intratracheally inoculated with *P. aeruginosa* PAO1, whereas control animals received PBS or AA alone. Forty-eight hours later, PAO1-infected mice were administered intraperitoneal gentamicin (7.5 mg/kg) and/or AA (2.74 mg/kg) according to the experimental grouping. For the intratracheal inoculation, anesthetized mice were positioned supinely, and the neck area was disinfected with 70% ethanol. A vertical incision was made to open the trachea, and 2 × 10^6^ CFU of *P. aeruginosa* in 50 µL PBS was injected. The incision was closed using a Skin Stapler (Visistat, USA), and mice were placed on a heating pad at 37 °C for about 15 min until recovery. At 48 h post-infection, infected mice were treated with intraperitoneal injections of gentamicin (7.5 mg/kg) and/or AA (2.74 mg/kg). Mice were euthanized 48 h after treatment, and lung tissues were harvested for analysis (see Figure 2 for the entire procedure).

Histological Analysis. Tissue samples were fixed in 4% neutral buffered formalin for 24 h, dehydrated through a graded ethanol series, and embedded in paraffin. The paraffin-embedded specimens were sectioned at 4-μm thickness using a microtome. Sections were deparaffinized, rehydrated, and stained with hematoxylin and eosin (H&E) and periodic acid-Schiff (PAS) [27] following standard protocols. Histological examination was performed using light microscopy (ECLIPSE, NIKON, Tokyo, Japan), and representative images were captured using imaging software (NIS-Elements Viewer, NIKON, Japan). To quantify the extent of pulmonary damage, semi-quantitative scoring systems were employed for tissue sections. For H&E staining, structural lung damage, inflammatory cell infiltration, and alveolar wall thickening were evaluated using a standardized 5-point lung injury scoring rubric: Score 0 (normal), Score 1 (mild), Score 2 (moderate), Score 3 (extensive), and Score 4 (severe injury including alveolar collapse). Airway mucus hypersecretion was evaluated in PAS-stained sections using a semi-quantitative mucus (PAS+) scoring system on a 0 to 4 scale, based on the extent and intensity of PAS-positive luminal mucus deposition (Score 0, absent/normal; Score 1, slight; Score 2, moderate; Score 3, severe; Score 4, extensive mucus occlusion). To minimize subjective bias, all scoring was performed by two independent researchers and further validated through two separate AI-based image analysis.

Organ Index. To calculate organ indices, the heart, liver, spleen and kidneys were dissected and weighed, and each organ index was calculated as organ weight (mg) divided by body weight (g).

RNA extraction and quantitative real-time PCR (RT-qPCR) analysis. Total RNA was extracted from mouse lung tissue (50 mg) using RiboEx^TM^ (GeneAll, Republic of Korea) according to the manufacturer’s instructions, dissolved in sterilized RNase-free water, and stored at −80 °C. cDNA was synthesized from 2 μg of the RNA using SuPrimeScript RT Premix (GeNet Bio, Daejeon, Republic of Korea) according to the manufacturer’s instructions. RT-qPCR was performed using cDNA, specific primers (Table 1) were designed in this study using Primer3 software and validated by NCBI Primer-BLASTn (BLAST+, version 2.17.0; National Center for Biotechnology Information, Bethesda, MD, USA) and the TB Green Premix Ex Taq Kit (TaKaRa Bio; Otsu, Japan) on a Thermal Cycler Dice Real-Time System TP800 (TaKaRa Bio; Otsu, Japan). To quantify the gene expression, the relative mRNA expression levels were calculated using the 2^−ΔΔCt^ method, with β-actin as the internal control for normalization. All data were expressed as fold-changes relative to the untreated control group (ΔΔCt = (C_t,target_ − C_t,β-actin_)_sample_ − (C_t,target_ − C_t,β-actin_)_untreated control_; Relative Fold Change = 2^−ΔΔCt^).

Statistical analysis. For significance of the results, the data were statistically analyzed using the *t*-test (two-sample assuming equal variances) in MS Office Excel for Mcrosoft Office Professional Plus 2019 (Microsoft, WA, USA). If the *p*-value was lower than 0.05, it was considered significant.

## 3. Results

### 3.1. In Vivo Evaluation of the Therapeutic Potential of Anthranilate, an Anti-Virulence Compound, in P. aeruginosa Skin Infection

To evaluate the therapeutic potential of anthranilate as an antibiotic adjuvant or a standalone against *P. aeruginosa* infection, a mouse skin infection model was first employed. In this mouse skin infection model, circular wounds were created on the dorsal surface for compromised skin barrier, and infection was established by inoculating *P. aeruginosa* on the wound sites (Figure 1). After 2 days, when the infection progressed to the point of causing slight suppuration at the wound, we administered anthranilate, and for comparison, gentamicin in two different ways: direct application to the infection site and intraperitoneal injection.

In the control group without *P. aeruginosa* infection (PBS), wounds showed normal healing progression and almost recovered in 8 days (Figure 3A). In contrast, *P. aeruginosa*-infected wounds without treatment (PAO1) exhibited significant deterioration with pronounced purulent discharge and inflammation confirming the severe establishment of *P. aeruginosa* infection (Figure 3A). To objectively assess these clinical observations, the infection severity and wound progression were semi-quantified using the pus score and relative wound area, as presented in Figure 3B. However, when anthranilate was directly applied to the infection site 2 days after infection (PAO1 + AA), the slight suppuration that had developed on the 2nd day ceased, and almost complete recovery was observed in 8 days (Figure 3A,B). For comparison, gentamicin (PAO1 + GM) applied directly to the wound site after infection also showed a similar level of healing effect as anthranilate (Figure 3A,B). When both gentamicin and anthranilate were administered together, they showed similar levels of therapeutic effect, indicating that there was minimal synergistic effect between the two substances (Figure 3A,B). While all treatments demonstrated good recovery effects in skin wounds with reduced infection severity and progression of healing, anthranilate alone also demonstrated a similar level of therapeutic effect. This indicates that anthranilate is sufficiently effective in inhibiting infection. Furthermore, anthranilate alone (AA) showed no adverse effects on wound healing, confirming its safety for topical application (Figure 3A,B).

To assess the systemic impact of *P. aeruginosa* infection and treatments, body weight changes were monitored throughout the experiment (Figure 3C). The control group and anthranilate-only group showed minimal initial weight loss with gradual recovery after Day 4. In contrast, the PAO1-infected group (PAO1) demonstrated continuous weight loss, maintaining approximately 9% reduction from initial body weight by Day 8 (Figure 3C). Notably, both the anthranilate- and gentamicin-treated groups (PAO1 + AA and PAO1 + GM) showed an accelerated recovery of body weight compared to the infected group, with the anthranilate-treated group exhibiting a modestly faster recovery (Figure 3C). In contrast, the combined treatment group (PAO1 + AA + GM) effectively reduced infection severity but displayed delayed weight recovery, likely due to the physiological burden associated with gentamicin administration (Figure 3C).

**Figure 4:**
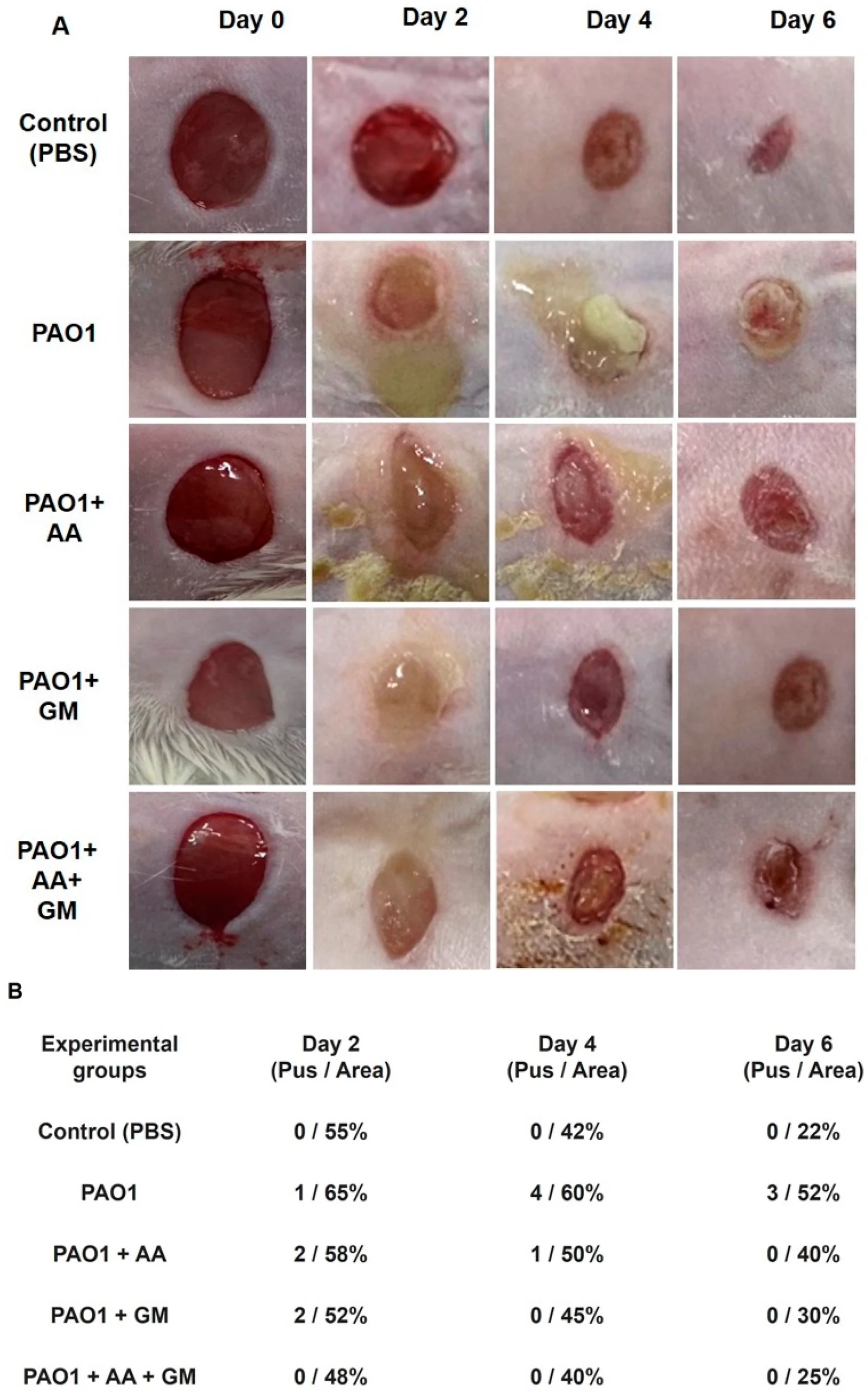
The effects of anthranilate on mouse skin infections—intraperitoneal administration. (**A**) The mouse skin infection was conducted in the same manner as the experiment described in Figure 3. However, AA (2.74 mg/kg) and/or GM (7.5 mg/kg) were administrated via intraperitoneal injection of 600 µL by syringe on the 2nd day after infection. *n* = 4–5 per each experimental group. (**B**) The infection severity and wound progression in (**A**) were quantified using the semi-quantitative pus score and the relative wound area (%), as described for Figure 3B.

Administering anthranilate via intraperitoneal injection also demonstrated a good recovery effect for treating *P. aeruginosa* skin infections (Figure 4A). The control group without *P. aeruginosa* infection (control) showed normal wound healing progression, and the PAO1-infected group without treatment (PAO1) showed progressive infection 2 days after infection (Figure 4A). To evaluate the systemic efficacy quantitatively, the severity of infection and wound closure were semi-quantified using the pus score and relative wound area, as presented in Figure 4B. However, the anthranilate treatment through intraperitoneal injection (PAO1 + AA) showed significant improvement with reduced wound size and markedly decreased inflammation (Figure 4A,B). This experiment also demonstrated that treatments with gentamicin alone and in combination with anthranilate resulted in similar levels of recovery as anthranilate treatment alone. This suggests that anthranilate can reach the local infection site and exert therapeutic effects even when administered systemically. This result indicates that anthranilate can effectively alleviate *P. aeruginosa* skin infections through intraperitoneal administration, suggesting its potential value as an alternative therapy to conventional gentamicin treatment.

### 3.2. In Vivo Evaluation of the Therapeutic Effects of Anthranilate in a Mouse Model of P. aeruginosa Lung Infection

Next, we evaluated the therapeutic efficacy of anthranilate in a mouse model of lung infection. To establish infection, *P. aeruginosa* was introduced directly into the trachea via a surgical incision to ensure consistent bacterial delivery. Two days after infection, anthranilate or gentamicin was administered intraperitoneally, and after another two days, the lungs were collected for histopathological and molecular analyses (see Figure 2).

**Figure 5:**
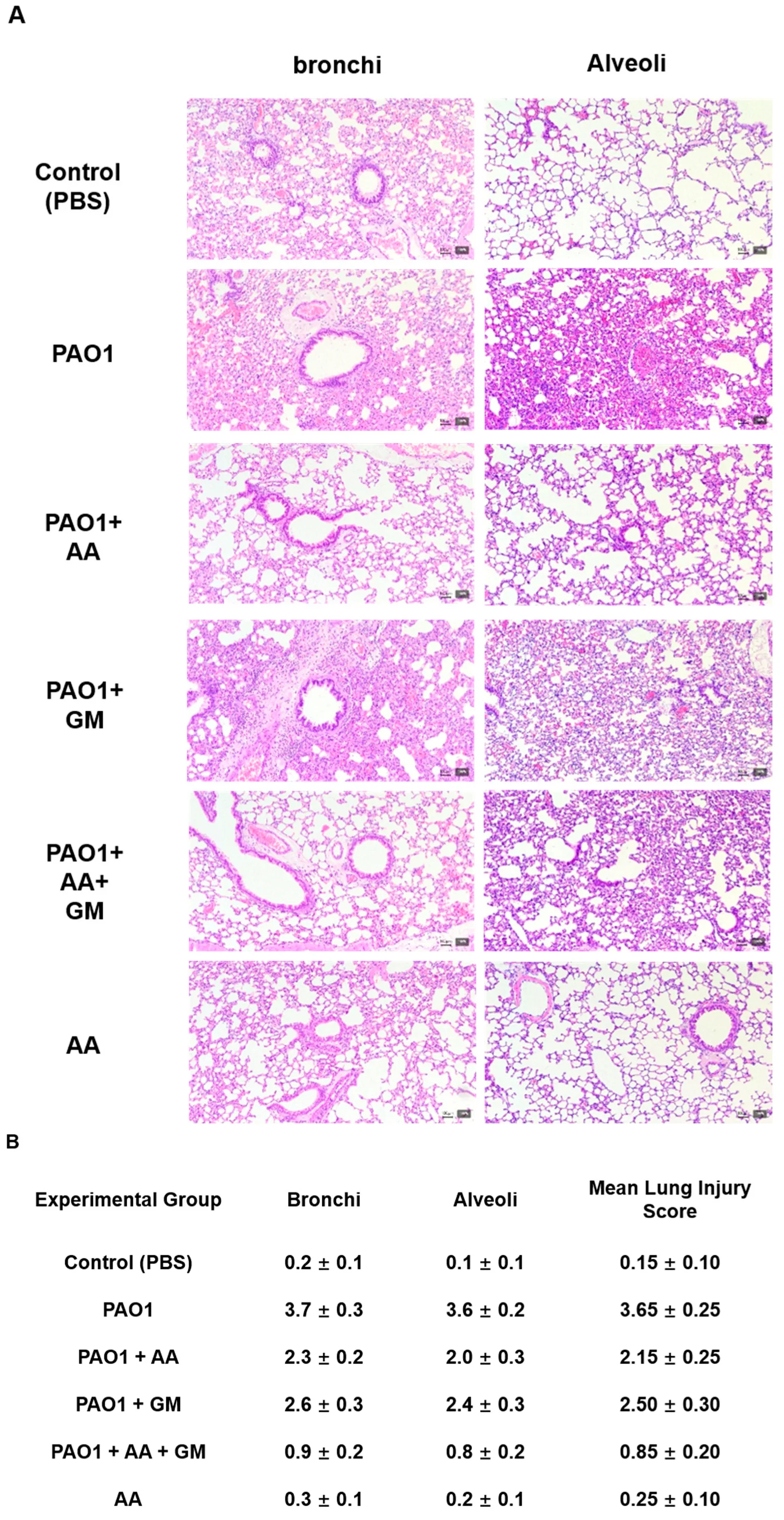
Histological observations of *P. aeruginosa* lung tissue—H&E staining. (**A**) After mouse lung infection and treatments, lung tissues were harvested and stained with H&E to evaluate histological changes. The experimental groups and the number of mice used were the same as those in the skin infection experiments. (**B**) Infection severity was quantified using a semi-quantitative lung injury scoring system (0–4) based on structural damage, inflammatory cell infiltration, and alveolar wall thickening.

To assess histological changes induced by infection and treatment, lung tissues were examined by H&E staining (Figure 5A). The tracheal and alveolar regions were the primary sites of observation, and pathological alterations were compared. In the uninfected control group (PBS), normal alveolar architecture, bronchi, and blood vessels were observed without any signs of inflammation or tissue injury. In contrast, the *P. aeruginosa*-infected group (PAO1) exhibited extensive tissue damage characterized by dense inflammatory cell infiltration, severe disruption of alveolar and bronchial structures, and perivascular hemorrhage (Figure 5A). These results confirmed the establishment of a severe lung infection. To provide a more objective evaluation, these histological changes were semi-quantified using a lung injury scoring system, as presented in Figure 5B. Treatment with anthranilate, gentamicin, or their combination partially alleviated these pathological alterations. In the treated groups (PAO1 + AA, PAO1 + GM, and PAO1 + AA + GM), inflammatory infiltration and structural disruption were noticeably reduced compared to the untreated infected group (Figure 5A,B). These findings indicate that anthranilate attenuates the severity of *P. aeruginosa*-induced lung injury.

**Figure 6:**
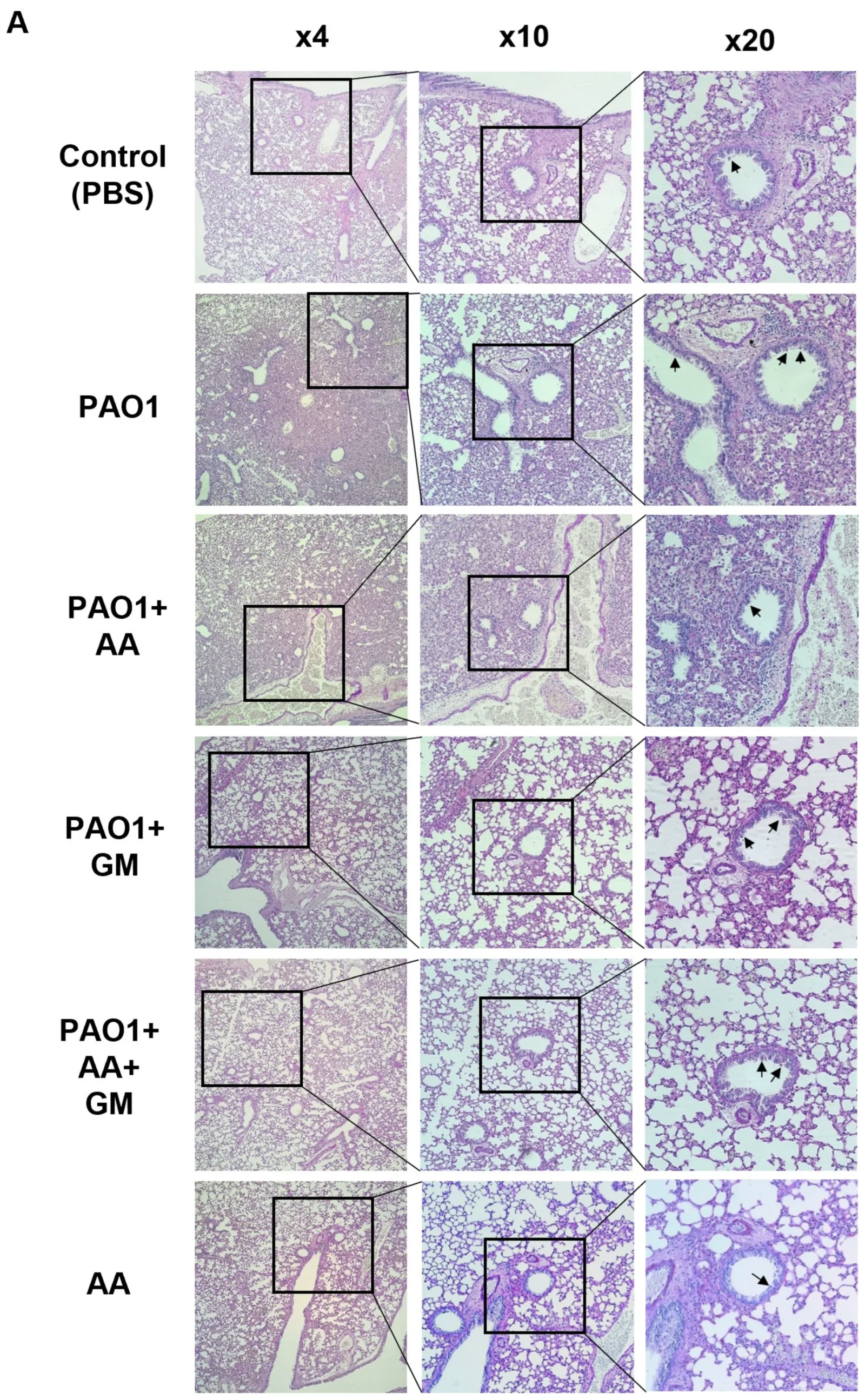

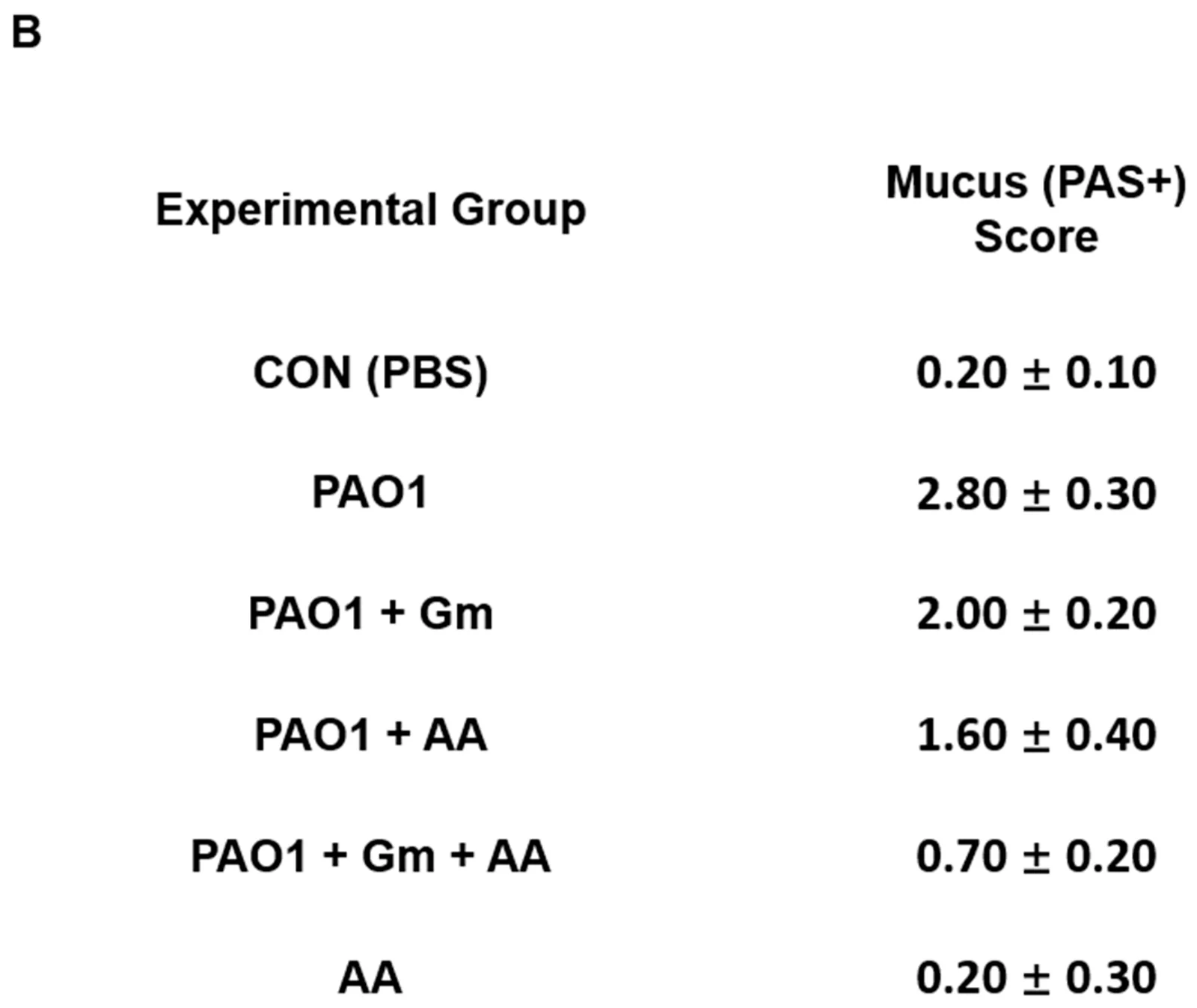
Histological observations of *P. aeruginosa* lung tissue—PAS staining. (**A**) Lung tissues were stained with PAS staining to examine histological changes. The experimental groups and the number of mice used were the same as those in the skin infection experiments. Arrows indicates PAS positive area. (**B**) Infection severity was quantified using a semi-quantitative mucus (PAS+) scoring system (0–4) based on extent and intensity of PAO-positive luminal mucus deposition.

To evaluate pulmonary mucus production and associated airway pathology, lung tissues were subjected to PAS staining (Figure 6A). In the uninfected control group (PBS), clear bronchial epithelial linings with minimal PAS-positive signals were observed (Figure 6B). Conversely, the *P. aeruginosa*-infected group (PAO1) exhibited an increase in PAS-positive material accumulating along the bronchial lumen, resulting in a significantly elevated score (Figure 6B). Treatment with anthranilate alone (PAO1 + AA) or gentamicin alone (PAO1 + GM) markedly reduced this PAS-positive accumulation. Notably, combination therapy (PAO1 + AA + GM) exerted an additive effect, further reducing PAS-positive mucus deposition. The anthranilate-alone group (AA) showed a baseline score comparable to the control, confirming its safety profile. These results demonstrate that anthranilate effectively suppresses *P. aeruginosa*-induced airway mucus hypersecretion, indicating that anthranilate treatment mitigates goblet cell metaplasia and hyperactive secretory responses in infected lung tissues.

### 3.3. Anthranilate Exerts Anti-Inflammatory Effects During P. aeruginosa Lung Infection Through the Suppression of Proinflammatory Cytokine Production

**Figure 7:**
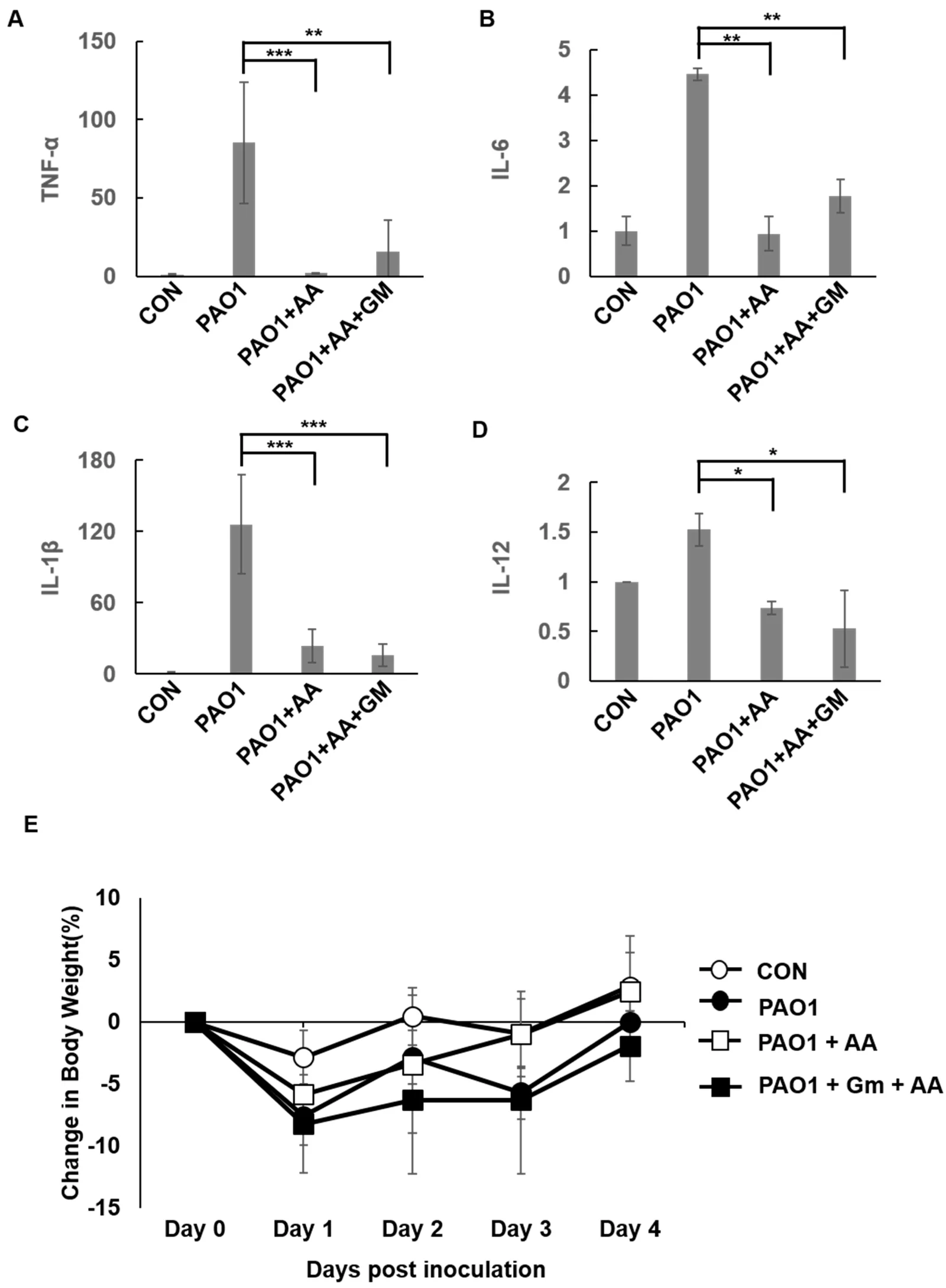
Expression of proinflammatory cytokines. The mRNA levels of the inflammatory cytokines, TNF-α (**A**), IL-6 (**B**), IL-1β (**C**), and IL-12 (**D**) were measured in the lung tissues using RT-qPCR. Cytokine mRNA expression levels were normalized to β-actin and are presented as relative fold-changes (2^−ΔΔCt^) compared to the untreated control group (set to 1). *, *p* < 0.05; **, *p* < 0.005; ***, *p* < 0.0005. (**E**) During the lung infection experiment, body weights were measured following the infection. Data were expressed as a percentage change from the initial weight.

The expression of proinflammatory cytokines, including IL-6, IL-12, IL-1β, and TNF-α, was quantified by RT-qPCR in lung tissues collected from *P. aeruginosa*-infected mice (Figure 7). Infection with *P. aeruginosa* markedly upregulated all four cytokines, and treatment with anthranilate alone or in combination with gentamicin significantly reduced their expression, approaching levels observed in the uninfected controls (Figure 7A–D). Notably, while IL-6, IL-1β, and TNF-α exhibited robust fold-increases upon infection, IL-12 showed a relatively modest ~1.5-fold induction compared to the uninfected controls, yet reached statistical significance (Figure 7D), likely attributable to the kinetic transition toward adaptive immunity as well as specific suppression by *P. aeruginosa* QS molecules, particularly 3-oxo-C12-HSL, as suggested by Skindersoe et al. [28]. Body weight changes were also monitored to evaluate systemic effects during infection and recovery. Although transient fluctuations were observed following infection, no significant differences were detected among treatment groups (Figure 7E).

### 3.4. Inhibitory Effect of Anthranilate on Pulmonary Bacterial Burden and Assessment of Systemic Toxicity

**Figure 8:**
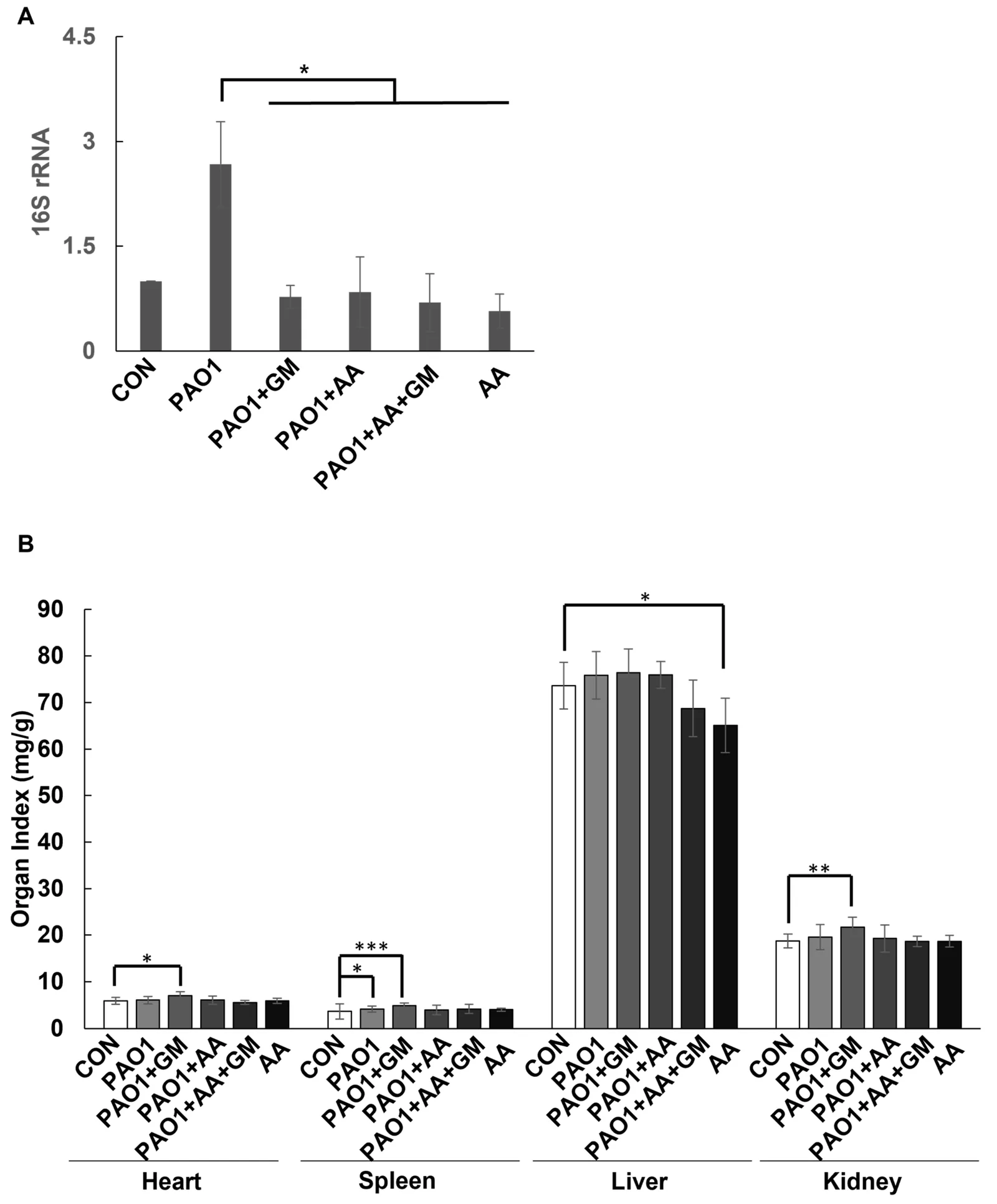
Expression of 16s rRNA and organ index was measured. (**A**) Bacterial 16S rRNA levels in lung tissues were measured using RT-qPCR and normalized to host β-actin expression. The results are presented as relative fold changes calculated using the 2^−ΔΔCt^ method, with the untreated control group set to 1. *, *p* < 0.05; **, *p* < 0.005; ***, *p* < 0.0005. (**B**) On Day 4, mice were sacrificed and organ indices of the heart, spleen, liver, and kidneys were calculated.

To evaluate the antibacterial efficacy of anthranilate in vivo, the bacterial burden in lung tissues was quantified by measuring *P. aeruginosa* 16S rRNA transcript levels using RT-qPCR (Figure 8A). Infection with *P. aeruginosa* (PAO1) resulted in a significant elevation of bacterial 16S rRNA levels in the lungs. In contrast, treatment with anthranilate alone (PAO1 + AA), gentamicin alone (PAO1 + GM), or their combination (PAO1 + AA + GM) significantly decreased bacterial 16S rRNA abundance compared to the untreated infected group (Figure 8A), demonstrating that anthranilate effectively promotes bacterial clearance from infected lung tissues.

Since the lungs were the direct site of pathogen inoculation and thus naturally subject to primary infectious and inflammatory changes, organ indices (organ weight to body weight ratio, mg/g) were evaluated in other major visceral organs—the heart, spleen, liver, and kidney—to assess secondary systemic toxicity and organ damage (Figure 8B). While gentamicin treatment (PAO1 + GM) induced slight fluctuations in organ index values in some tissues, anthranilate treatment—both alone (AA) and in combination with infection (PAO1 + AA)—showed no discernible alterations in the heart, spleen, or kidney compared to the uninfected control [29], with minor variation limited only to the liver (Figure 8B). These results highlight that anthranilate effectively clears pulmonary infection while possessing a favorable systemic safety profile with lower visceral toxicity risk compared to conventional gentamicin treatment.

## 4. Discussion

*P. aeruginosa* is a major opportunistic pathogen responsible for severe acute and chronic infections and is characterized by a high propensity for antibiotic resistance [30,31]. Consequently, there is an urgent need for alternative therapeutic strategies that can control infection while minimizing selective pressure for resistance development [32]. In this study, we evaluated anthranilate as a potential anti-virulence agent and demonstrated its efficacy in mitigating *P. aeruginosa* infections without directly inhibiting bacterial growth. Our findings show that anthranilate significantly alleviated pathological manifestations of *P. aeruginosa* infection in both skin and lung infection models. Treatment reduced tissue damage, inflammatory responses, and purulent discharge while promoting wound recovery and restoring tissue integrity. Notably, the therapeutic outcomes achieved with anthranilate were comparable to those observed with gentamicin, despite the absence of bactericidal or bacteriostatic activity. These results indicate that anthranilate effectively suppresses *P. aeruginosa* pathogenesis by attenuating virulence rather than eradicating the bacteria. Therefore, it serves as evidence that anthranilate works well as an anti-virulence agent.

In this study, we sought to ensure maximum objectivity and transparency by integrating comprehensive visual evidence with quantitative and semi-quantitative assessments. For the skin infection model, we presented sequential images of the entire healing process, supplemented by both semi-quantitative pus scoring and quantitative, AI-based wound area measurements. Similarly, the efficacy in the lung infection model was demonstrated through histological sections of multiple tissues, including the trachea and alveoli, which were further supported by semi-quantitative lung injury scoring. We believe that this combined approach—prioritizing the direct visualization of stage-by-stage recovery alongside complementary scoring—provides a more intuitive and biologically representative reflection of therapeutic ‘phenotypic rescue’.

Anthranilate functions to downregulate virulence factors and biofilm formation, thereby reducing bacterial pathogenicity while preserving bacterial viability [18]. This mode of action represents a potential advantage over conventional antibiotics. In theory, since anthranilate does not impose lethal or growth-inhibitory pressure, it is hypothesized to exert lower selective pressure to drive the emergence of antibiotic resistance. However, it is important to acknowledge that resistance to anti-virulence agents can still emerge through mutations in regulatory genes or efflux pumps, as suggested in recent literature [33]. While we did not experimentally evaluate long-term resistance development through repeated exposure in this study, the maintenance of bacterial viability may facilitate clearance by host immune mechanisms, supporting infection resolution while minimizing evolutionary pressure for resistant mutants. These features underscore the potential of anthranilate as a therapeutic option, warranting further investigation regarding its long-term resistance profile, particularly for chronic or recurrent *P. aeruginosa* infections.

The concept underlying anthranilate’s activity aligns with the broader paradigm shift from traditional bactericidal therapies toward anti-virulence strategies. While classical antimicrobial development has historically focused on increasing antibiotic potency—often accelerating resistance—anti-virulence approaches aim to disarm pathogens without inhibiting growth [11]. More recently, the anti-pathogenic framework has expanded this concept to include the modulation of host responses, such as limiting excessive inflammation or enhancing immune-mediated clearance [34]. Anthranilate fits well within this framework, as it suppresses bacterial virulence while simultaneously reducing infection-associated inflammation and tissue damage. Importantly, anthranilate exhibited a favorable safety profile in vivo. Neither topical nor systemic administration caused detectable toxicity, as evidenced by normal tissue histology and stable body weight in the anthranilate-treated animals. These observations support the feasibility of anthranilate for both local and systemic applications.

In conclusion, this study highlights anthranilate as a promising anti-virulence and anti-pathogenic therapeutic candidate capable of suppressing *P. aeruginosa* infection by modulating virulence and host–pathogen interactions rather than bacterial survival. By avoiding direct bactericidal pressure, anthranilate may represent a potentially resistance-sparing alternative or complement to conventional antibiotics, although the long-term risk of resistance emergence remains to be fully characterized. Future studies aimed at elucidating its molecular mechanisms and expanding its application across diverse pathogens will further clarify its potential role in combating antibiotic-resistant infections.

## Figures and Tables

**Table 1:** Primers used in this study.

Primers	Forward/Reverse (5′–3′)	References
β-actin	TCACACACTGTCCCCATCTACG /ACCACGCTCGGTCAGGATTTTC	[26]
TNF-α	CATCTTCTCAAAATTCGAGTGACAA /TGGGAGTAGACAAGGTACAACCC	[26]
IL-1β	GCCCATCCTCTGTGACTCAT /AGGCCACAGGTATTTTGTCG	[26]
IL-6	CAGAATTGCCATCGTACAACTCTTTTCTCA /AAGTGCATCATCGTTGTTCATACA	[26]
IL-12	GGAAGCACGGCAGCAGAATA /AACTTGAGGGAGAAGTAGGAATGG	[26]
16s rRNA	CGGACCTCACGCTATCAGAT /CTGCCCTTCCTCCCAACTTA	This work

## Data Availability

The original contributions presented in this study are included in the article. Further inquiries can be directed to the corresponding author(s).
